# Linking Aboveground Traits to Root Traits and Local Environment: Implications of the Plant Economics Spectrum

**DOI:** 10.3389/fpls.2019.01412

**Published:** 2019-10-30

**Authors:** Yong Shen, Gregory S. Gilbert, Wenbin Li, Miao Fang, Huanping Lu, Shixiao Yu

**Affiliations:** ^1^Department of Ecology, School of Life Sciences/State Key Laboratory of Biocontrol, Sun Yat-sen University, Guangzhou, China; ^2^Department of Environmental Studies, University of California, Santa Cruz, Santa Cruz, CA, United States; ^3^Guangdong Ecological Meteorology Center, Guangzhou, China

**Keywords:** functional traits, light, plant economics spectrum, soil fertility, subtropical forest, woody seedling

## Abstract

The plant economics spectrum proposes that ecological traits are functionally coordinated and adapt along environmental gradients. However, empirical evidence is mixed about whether aboveground and root traits are consistently linked and which environmental factors drive functional responses. Here we measure the strength of relationships between aboveground and root traits, and examine whether community-weighted mean trait values are adapted along gradients of light and soil fertility, based on the seedling censuses of 57 species in a subtropical forest. We found that aboveground traits were good predictors of root traits; specific leaf area, dry matter, nitrogen and phosphorus content were strongly correlated with root tissue density and specific root length. Traits showed patterns of adaptation along the gradients of soil fertility and light; species with fast resource-acquisitive strategies were more strongly associated with high soil phosphorus, potassium, openness, and with low nitrogen, organic matter conditions. This demonstrates the potential to estimate belowground traits from known aboveground traits in seedling communities, and suggests that soil fertility is one of the main factors driving functional responses. Our results extend our understanding of how ecological strategies shape potential responses of plant communities to environmental change.

## Introduction

Functional traits can be powerful indicators of the ecological strategies of plant species (Wright et al., 2004; Freschet et al., 2010; de la Riva et al., 2016), and trait-based approaches have provided important insights toward understand what drives the structure of biological communities (Messier et al., 2017; Umana et al., 2017). One significant achievement in trait-based ecology is the identification of an economics spectrum of plant traits that helps conceptually organize trade-offs between resource acquisition and conservation (Wright et al., 2004; Chave et al., 2009; Weemstra et al., 2016). The plant traits associated with the leaf economics spectrum (LES) and wood economics spectrum (WES) tend to be coordinated; there is often a one-dimensional trade-off gradient from resource conservative to resource acquisitive strategies (Wright et al., 2004; Chave et al., 2009; Pivovaroff et al., 2014). However, in part because root traits are more difficult to collect than leaf and wood traits, the root economics spectrum (RES) is much less well understood than its aboveground counterparts (Weemstra et al., 2016; Bergmann et al., 2017). Plant economics spectrum (PES) theory attempts to integrate leaf, stem, and root traits to explain plant ecological strategies (Reich, 2014; Diaz et al., 2016). It predicts that (1) the leaf, stem, and root traits are correlated with each other and coordinated across different organs, and (2) functional traits should have adaptive significance along resource gradients. This leads to two inferences: (1) because all organs should be consistently resource-acquisitive or resource-conservative for all resources, readily measured aboveground traits should be useful predictors of root traits that are more difficult to measure; and (2) if traits from different organs are coordinated, then they should response to environmental gradients consistently (e.g., fast-growing, resource-acquisitive species should be more abundant in resource-rich environments, and slow-growing, resource-conservative species should be more abundant in resource-poor environments) (Reich, 2014; Kramer-Walter et al., 2016).

The hypothesis of trait coordination has general support. For example, plant traits regulating photosynthetic and hydraulic capacity are coordinated, and those traits are also related to anatomical, leaf, and stem traits (Santiago et al., 2004; Aasamaa et al., 2005; Taylor and Eamus, 2008). Traits are significantly correlated across leaves, stems, and roots in a subarctic flora (Freschet et al., 2010), in Mediterranean forests and shrublands (de la Riva et al., 2016), and in a Mediterranean rangeland (Perez-Ramos et al., 2012). However, some studies did not support consistent coordination between aboveground and belowground traits. For instance, Fortunel et al. (2012) found that root traits were closely aligned with stem traits but not with leaf traits across 758 Neotropical tree species (Fortunel et al., 2012), and Kramer-Walter et al. (2016) reported that although many root, stem, and leaf traits of tree seedlings were coordinated, specific root length was not (Kramer-Walter et al., 2016). Recent studies suggest that because roots face a more complex environment than aboveground organs (Weemstra et al., 2016), root traits are more multidimensional than those of leaves, so a single acquisition-conservation axis cannot adequately capture the variety of belowground functions and tradeoffs that drive differences in plant performance across species (Kramer-Walter et al., 2016; Weemstra et al., 2016; Bergmann et al., 2017; Shen et al., 2019). In addition, because the relationships between leaf and root traits can shift depending on soil properties (Craine et al., 2005), water availability (Fort et al., 2013), temperature (Geng et al., 2014), and other environmental conditions, it is unclear whether aboveground traits can be used reliably to predict belowground plant functions.

**Figure 1:**
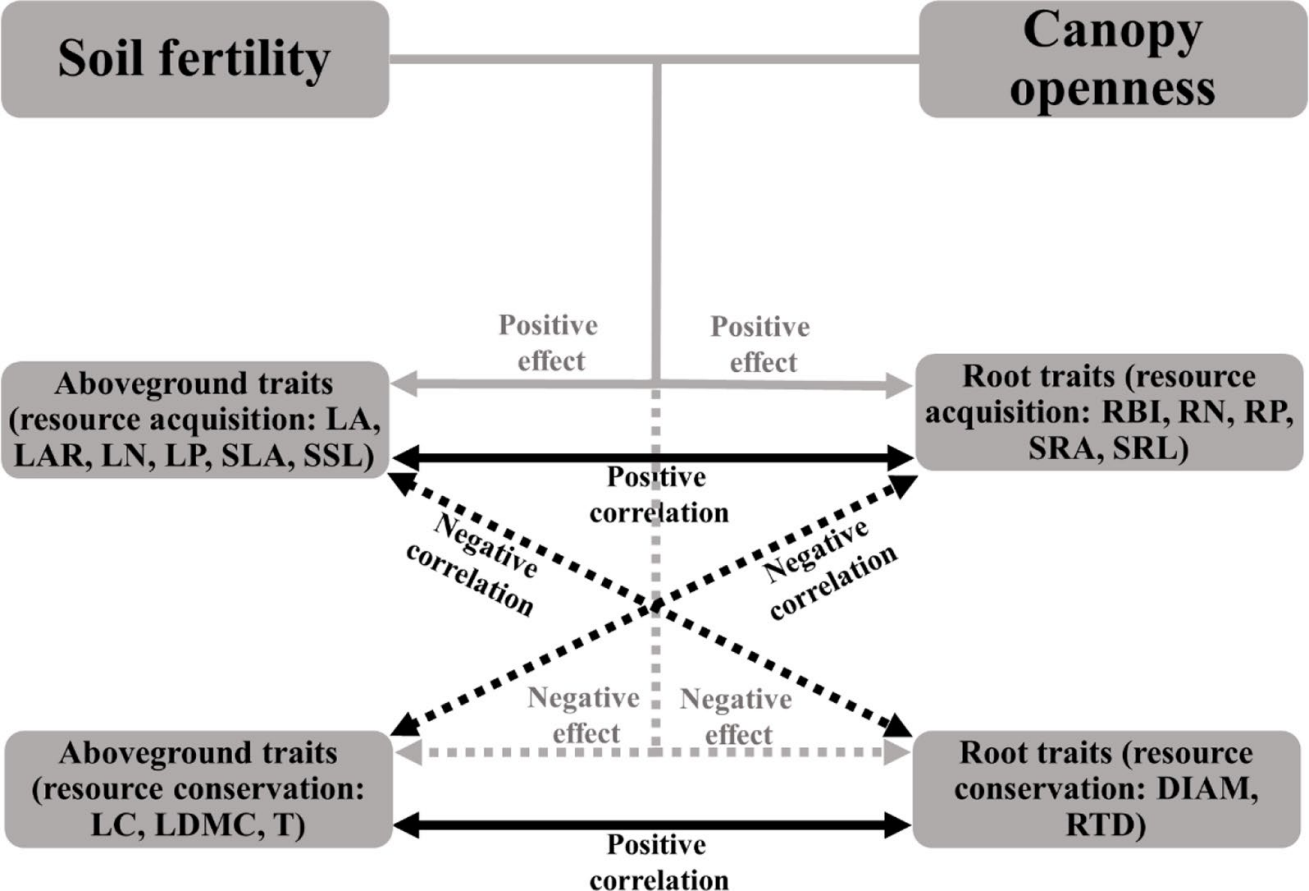
Illustration of the expected relationships among soil fertility, canopy openness, aboveground traits, and root traits based on the plant economic spectrum. Solid arrows represent positive effects or correlations, dashed arrows represent negative effects or correlations. Grey arrows indicate the effects of environmental conditions on traits, black arrows indicate the relationships between aboveground and root traits. See **Table 1** for trait abbreviations.

If the plant economics spectrum is a robust feature of plant communities, traits from different organs should be coordinated within species, and should show adaptive patterns along gradients of associated resources. We would expect, for example, that plants in canopy gaps and areas of fertile soil should have a fast, resource-acquisitive strategy; that is, species should have high specific leaf area (SLA), specific root length (SRL), leaf and root nitrogen (LN, RN), and have traits associated with low tissue density (e.g., low leaf dry matter content, root tissue density) (see **Figure 1** for details) (Poorter et al., 2008; Martinez-Vilalta et al., 2010; Wright et al., 2010; Mommer et al., 2011; Hajek et al., 2013; Weemstra et al., 2016). In contrast, slow-growing species with a resource-conservative strategy (with traits contrasting to those above) should be more abundant in environments with low light and limiting soil nutrients (Reich, 2014; Kramer-Walter et al., 2016). However, empirical studies found that these associations were inconsistent. For example, Freschet et al. (2010) reported that resource acquisitive species tended to grow in the soils with high N and litter water content (Freschet et al., 2010); High leaf and litter quality (e.g., high N) were associated with high N availability in soil, while high root quality did not show the same relationships to soil properties (Orwin et al., 2010). Kramer-Walter et al. (2016) found that root traits other than tissue density were not coordinated with variation in aboveground traits along a soil fertility gradient (Kramer-Walter et al., 2016). There is a critical need for further studies that simultaneously consider belowground and aboveground traits along environmental gradients to test the prediction of the plant economics spectrum for coordinated variation in whole-plant traits.

**Table 1:** The definition, abbreviation, and unit for 16 functional traits in this study.

	Trait	Abbreviation	Definition	unit
Aboveground trait	Leaf area	LA	Leaf surface area	cm^2^
	Leaf area ratio	LAR	The ratio of total leaf area to biomass of an individual	cm^2^ g^-1^
	Leaf carbon content	LC	Leaf carbon content per mass	%
	Leaf dry matter content	LDMC	The ratio of leaf dry mass to leaf fresh mass	g g^-1^
	Leaf nitrogen content	LN	Leaf nitrogen content per mass	mg g^-1^
	Leaf phosphorus content	LP	Leaf phosphorus content per mass	mg g^-1^
	Specific leaf area	SLA	Dividing leaf area by dried mass	cm^2^ g^-1^
	Specific stem length	SSL	The ratio of stem length to stem dry weight	cm g^-1^
	Leaf thickness	T	Leaf thickness at the widest part of each leaf	cm
Root trait	Fine-root diameter	DIAM	Average fine root diameter	mm
	Root branching intensity	RBI	Number of root tips per fine-root length	tips cm^-1^
	Root nitrogen content	RN	Root nitrogen content per mass	mg g^-1^
	Root phosphorus content	RP	Root phosphorus content per mass	mg g^-1^
	Root tissue density	RTD	The ratio of root dry mass to its volume	g cm^-3^
	Specific root area	SRA	The root surface area per dry mass	cm^2^ g^-1^
	Specific root length	SRL	The root length divided by its dry mass	cm g^-1^

To address this need, we combined information on woody plant censuses of 17,148 seedlings of 57 common species across 10 years with seedling functional traits of roots, stems, and leaves, to examine the relationships between aboveground and belowground traits, and to examine whether these traits show patterns of adaptation along environmental gradients of light (canopy openness) and soil fertility in a typical subtropical forest in Southern China. Specifically, we addressed the following two questions: 1) How well do aboveground traits of 57 subtropical tree seedling species serve as indicators for corresponding root traits? 2) Do traits have adaptive associations along the gradients of soil fertility and light? Specifically, are seedling species with fast resource-acquisitive traits associated with environments of high light and soil fertility, and resource-conservative seedling species associated with resource-poor local environments?

## Materials and Methods

### Study Site

This study was conducted in Heishiding Nature Reserve (23°27′ N, 111°53′ E) in Guangdong Province, Southern China. Our study site is a subtropical evergreen broad-leaved forest, with a characteristic subtropical moist monsoon climate with two marked seasons (dry vs. wet). The average annual precipitation is about 1744 mm, most of which occurs between April and September (wet season). The average annual temperature is 19.6°C and the average monthly temperature ranges from 10.6°C in January to 28.4°C in July (Liang et al., 2015). The topography of the area is highly heterogeneous and the elevation of the Nature Reserve ranges from 150 to 930 m.

### Seedling Surveys

Six 1-ha permanent plots were established during winter 2007 to spring 2008 in our study site. Three plots are located at relatively high elevation (600 m above sea level) and the other three at lower elevation (340 m). All woody individuals with a diameter at breast height (DBH; measured at 1.3 m above the soil) ≥1 cm were measured, mapped, tagged, and identified to species. In each 1-ha plot, 200 1-m^2^ seedling plots were established to monitor early stage seedling dynamics. Plots were arranged in sets of four placed in a checkerboard pattern of alternate 10 × 10 m quadrants (**Figure S1**). All seedlings of woody plants (DBH <1 cm) have been surveyed every year from 2008 through 2017 (Liu et al., 2012), with new recruits added to the census each year as they appear.

### Soil Fertility and Light Measurements

Each 1-ha plot was divided into a grid of 100 10 × 10 m quadrants (**Figure S1**). In 2017, we collected 600 samples (0–10 cm depth) of topsoil across the six 1-ha permanent plots (100 soil samples per plot). To capture fine scale variation in soil fertility, we first assigned a fixed sample point in every other 10 × 10 m quadrant in a checkerboard pattern, for a total of 50 fixed sample points. We then randomly selected one fixed point from each row of five fixed points. At each of those 10 selected points, a compass direction (N, E, S, W, NE, NW, SE, or SW) was randomly selected, and an additional five soil samples were collected at 2 cm, 8 cm, 1 m, 2 m, and 8 m from the selected fixed point, resulting in an additional 50 sample points per ha (**Figure S2**). Seven soil fertilities, including organic matter (mg g^-1^), total and available content (mg g^-1^) of nitrogen (N), phosphorus (P), and potassium (K) were measured in our study. Soil fertilities were interpolated to each seedling plot in each 1-ha permanent plot (**Table S1**) using ordinary Kriging by “geoR” package in R (R Development Core Team, 2017). Seedling plots that were destroyed by natural and human factors, or that lacked our target species were removed, leaving 1158 seedling plots to include in our final analysis.

In addition, we assessed light conditions in the understory by measuring canopy openness (Record et al., 2016). Hemispherical photographs were taken on days with uniformly overcast sky at the center of each 1-m^2^ seedling plot with a digital camera (Nikon COOLPIX 4500) between March and April in 2017. Next, we used WinSCANOPY (Regent Instruments, Version 2005a) to obtain the percentage of the total hemisphere that was open to the sky (openness) as an integrated measure of light resources availability for each of the seedling plots (**Table S1**).

### Plant Traits

Seedlings (4–5 individuals per species, height ranges from 20 to 50 cm) were collected for dominant seedling species (those species with at least 10 individuals in the seedling survey) around the permanent plots in the Heishiding Nature Reserve in October 2017 to measure seedling traits. Individual plants collected within a species were separated by at least 20 m.

For leaf traits, we selected three relatively young, healthy, and fully expanded leaves from each seedling. After cleaning the leaf surface, samples were scanned using a flatbed scanner (EPSON V370, China) and ImageJ (version 1.43u, USA) software was used to measure leaf area (LA, cm^2^). Leaf thickness (T, cm) was determined using a micrometer, the average of one measurement on each side of the main vein (at the widest part and avoiding large secondary veins). The fresh mass of leaves was determined using a balance; leaf samples were then dried in an oven at 60°C for at least 48 h and then re-weighed to determine leaf dry mass. Specific leaf area (SLA, cm^2^ g^-1^) was calculated by dividing leaf area by its dried mass. Leaf dry matter content (LDMC, %) was calculated as the ratio of leaf dry mass to its fresh mass (Shen et al., 2014; Shen et al., 2016). Leaf carbon content (LC, %) was measured by potassium dichromate volumetric method, nitrogen content (LN, mg g^-1^), and phosphorus content (LP, mg g^-1^) were determined using the micro-Kjeldahl digestion followed by colorimetric determination on a flow injection auto-analyzer (Chen et al., 2016). Biomass allocation traits: leaf area ratio (LAR, cm^2^ g^-1^) was determined as the ratio of total leaf area to biomass of a seedling (sum of the dry weight of leaves, stems, and roots). Stem specific length (SSL, cm g^-1^) was calculated as the ratio of stem length (from base to tip of stem) to stem dry weight (not including leaves and branches) (**Table S2**). These leaf and biomass allocation traits are referred as “aboveground traits”.

For root traits, we selected 1-3 fine root branches (non-woody fine root with diameter <2 mm) with intact terminal branch orders (Kubisch et al., 2015; Liese et al., 2017). Next, roots were washed carefully, spread out in a water bath and scanned (EPSON STD4800, USA). We used a needle to separate overlapping roots if needed. We used WinRhizo 2013e software (Régent Instruments Inc., Canada) to analyze these images to obtain average root diameter (DIAM, mm), root length, surface area, and volume (Kubisch et al., 2015; Erktan et al., 2016; Liese et al., 2017; Weemstra et al., 2017). After scanning, roots were placed in an oven at 70°C for at least 72 h until constant weight to determine their dry mass. Root tissue density (RTD, g cm^-3^) was calculated as the ratio of root dry mass to its volume assuming that a root was a cylinder. Specific root length (SRL, cm g^-1^) was calculated as the root length divided by its dry mass (Kong et al., 2014). Specific root area (SRA, cm^2^ g^-1^) was calculated as root surface area per dry mass (Hajek et al., 2013). Root branching intensity (RBI, tips cm^-1^) was calculated as number of root tips per root length (Liese et al., 2017). Nitrogen content (RN, mg g^-1^) and phosphorus content (RP, mg g^-1^) were determined using the same methods used for leaves (**Table S2**).

### Data Analysis

In order to examine how well aboveground traits can be used to predict root traits (question 1), we performed univariate and multivariate phylogenetic generalized least squares (PGLS) models for aboveground and root traits by fitting a Brownian Motion model of character evolution (Martinez-Vilalta et al., 2010), using the function “pgls” in the “caper” package (Orme, 2013) in R 3.4.0. The PGLS model accounted for the phylogenetic effects between plant species within the linear model, which can avoid phylogenetic dependency between plant species due to shared evolutionary history (Martinez-Vilalta et al., 2010; Gerz et al., 2018), through calculating phylogenetic correlation that varies between 0 (phylogenetic independence) and 1 (traits covary in direct proportion to their shared evolutionary history) (**Table S3**). The phylogenetic correlation measures whether data show phylogenetic dependence, and incorporating this phylogenetic information improves the accuracy of phylogenetic analyses (Freckleton et al., 2002). We used Phylomatic (ver. 3, http://phylodiversity.net/phylomatic/) to construct the phylogenetic tree of the plant species (**Figure S3**), and phylocom (ver. 4.2) to add branch lengths (Pivovaroff et al., 2014). For the multivariate PGLS models, we combined all aboveground traits to predict each root trait, and used model selection method to establish optimal models based on Akaike information criterion (AIC). In the model selection, we established all candidate models by different aboveground trait combinations, and then used AIC to find an optimal model for predicting each root trait. Model selection was conducted using the “dredge” function in “MuMIn” package. Traits were standardized by subtracting the mean and dividing by the standard deviation so that all traits were equally weighted in fitting the models (Paine et al., 2012).

To test the associations between traits and local environments (question 2), the community-weighted mean (CWM) trait values for each trait were estimated to represent dominant trait values of each seedling plot. CWM trait values were calculated as the mean trait value weighted by seedling species abundance in each seedling plot (Garnier et al., 2004):

$$\text{CWM}=\sum_{i=1}^{n}P_{i}\times {\mathrm{trait}}_{i}$$

where *n* is the species number of a seedling plot; *P_i_* is the relative abundance of species *i* (total abundance from 2008 to 2017 in each seedling plot was used), and *trait_i_* is the trait value of species *i*. CWM was calculated for each seedling plot. CWM represents the community structure (abundance of species) and dominant trait value (e.g., low or high) of each seedling plot (Dominguez et al., 2012), and also allow to link to their environmental conditions (de la Riva et al., 2016; Kramer-Walter et al., 2016).

Because soil fertility measures are often highly correlated, we performed a principal component analysis (PCA) on all soil variables. The first principal component (PC1) explained 60.39% variation in soil fertility (**Table S4**), so that PC1 was used as a composite surrogate for soil fertility in further analysis.

Next, we tested the relationships between soil fertility (PC1), canopy openness, and plant community (CWM) traits to determine whether traits associated with fast resource acquisition are more associated with the environments of high light availability and soil nutrients where they should be better adapted (Kramer-Walter et al., 2016). We first established a univariate linear regression model for each environmental factor and trait, and then used a redundancy analysis (RDA) to integrate all environmental factors and CWM traits in each seedling plot (Block and Meave, 2017); this allowed us to examine inter-correlations of traits, and the relationships among soil fertility, openness and traits simultaneously. RDA was performed using the “rda” function in the “vegan” package (Oksanen et al., 2018).

## Results

### Aboveground Traits Extrapolate Root Traits

Aboveground traits were good predictors of some key root traits in the univariate PGLS models (**Table 2**), particularly for root tissue density (RTD), specific root area (SRA), and specific root length (SRL), since we found that most of the aboveground traits, such as LAR, LDMC, LN, LP, SLA, and SSL were correlated with each of them. The relationships between aboveground and root traits were consistent in sign. For example, traits that were positively associated with resource acquisition, such as LAR, SLA, LN, LP, SRA, and SRA, were significantly positively correlated with each other; traits that were positively associated with resource conservation, e.g. LDMC and RTD, were also positively correlated, and they had negative relationships with the traits that were positively connected to resource acquisition (see the expected relationships in **Figure 1**). In the optimal PGLS model, we found the best predictors (aboveground traits) for each of the root traits (**Table 3**). For example, leaf nitrogen content (LN) was the best predictor of root nitrogen content (RN); LAR and LN can best predict SRA. We captured a relatively large proportion of variation in root traits in the optimal models, especially for the root traits in the plant economics spectrum (RN, RP, RTD, SRA, and SRL), with R^2^ ranging from 0.22 to 0.40 using 1–3 predictors.

**Table 2:** Coefficient (P value and R^2^) of univariate phylogenetic generalized least squares models (PGLS) for root traits and aboveground traits of seedling species.

	DIAM	RBI	RN	RP	RTD	SRA	SRL
LA	-0.13 (0.13, 0.02)	0.10 (0.26, 0.01)	0.05 (0.62, 0.00)	0.16 (0.14, 0.02)	-0.02 (0.88, 0.00)	0.03 (0.82, 0.00)	-0.01 (0.93, 0.00)
LAR	***-0.03 (0.76, 0.00)***	-0.03 (0.77, 0.00)	0.20 (0.06, 0.04)	***0.36 (< 0.001, 0.17)***	***-0.43 (< 0.001, 0.22)***	***0.57 (< 0.001, 0.31)***	***0.48 (< 0.001, 0.24)***
LC	*0.22 (0.04, 0.06)*	-0.17 (0.15, 0.02)	0.06 (0.62, 0.00)	0.10 (0.47, 0.00)	-0.02 (0.90, 0.00)	0.00 (0.99, 0.00)	-0.19 (0.20, 0.01)
LDMC	0.12 (0.26, 0.01)	-0.13 (0.29, 0.00)	-0.23 (0.07, 0.04)	-0.26 (0.07, 0.04)	***0.35 (0.01, 0.11)***	***-0.41 (< 0.001, 0.14)***	***-0.38 (0.01, 0.11)***
LN	-0.07 (0.55, 0.00)	-0.03 (0.78, 0.00)	***0.52 (< 0.001, 0.26)***	0.07 (0.65, 0.00)	***-0.43 (0.00, 0.16)***	***0.45 (< 0.001, 0.19)***	***0.56 (< 0.001, 0.22)***
LP	-0.07 (0.50, 0.01)	0.17 (0.14, 0.02)	***0.29 (0.02, 0.08)***	0.14 (0.33, 0.00)	***-0.37 (0.01, 0.11)***	***0.43 (< 0.001, 0.17)***	***0.28 (0.04, 0.05)***
SLA	-0.02 (0.89, 0.00)	0.14 (0.23, 0.01)	0.21 (0.10, 0.03)	***0.33 (0.01, 0.10)***	***-0.35 (0.01, 0.10)***	***0.41 (0.00, 0.15)***	***0.31 (0.03, 0.06)***
SSL	0.04 (0.63, 0.00)	-0.08 (0.38, 0.00)	0.11 (0.31, 0.00)	0.16 (0.11, 0.03)	***-0.31 (0.01, 0.11)***	***0.32 (0.01, 0.09)***	***0.31 (0.01, 0.09)***
T	-0.02 (0.80, 0.00)	-0.04 (0.61, 0.00)	-0.14 (0.21, 0.01)	-0.16 (0.12, 0.03)	0.21 (0.09, 0.04)	***-0.29 (0.03, 0.07)***	-0.18 (0.16, 0.02)

Statistically significant relationships were highlighted in bold and italic (P < 0.05). See **Table 1** for trait abbreviations.

**Table 3:** Optimal multivariate phylogenetic generalized least squares models (PGLS) for root traits and aboveground traits of seedling species. Coefficients (and P values) are shown.

	DIAM	RBI	RN	RP	RTD	SRA	SRL
LA	-0.13 (0.11)			***0.20 (0.04)***			
LAR				***0.38 (< 0.001)***	***-0.35 (0.00)***	***0.47 (< 0.001)***	***0.29 (0.00)***
LC	***0.22 (0.04)***						***-0.28 (0.02)***
LDMC		-0.21 (0.12)					
LN		-0.28 (0.05)	***0.52 (< 0.001)***		***-0.32 (0.01)***	***0.28 (0.02)***	***0.53 (< 0.001)***
LP		***0.29 (0.03)***					
SLA							
SSL							
T							
R^2^	0.09	0.07	0.26	0.22	0.30	0.37	0.40

Statistically significant relationships were highlighted in bold and italic (P < 0.05). See **Table 1** for trait abbreviations.

### The Relationships Between Soil Fertility, Canopy Openness and Traits

**Figure 2:**
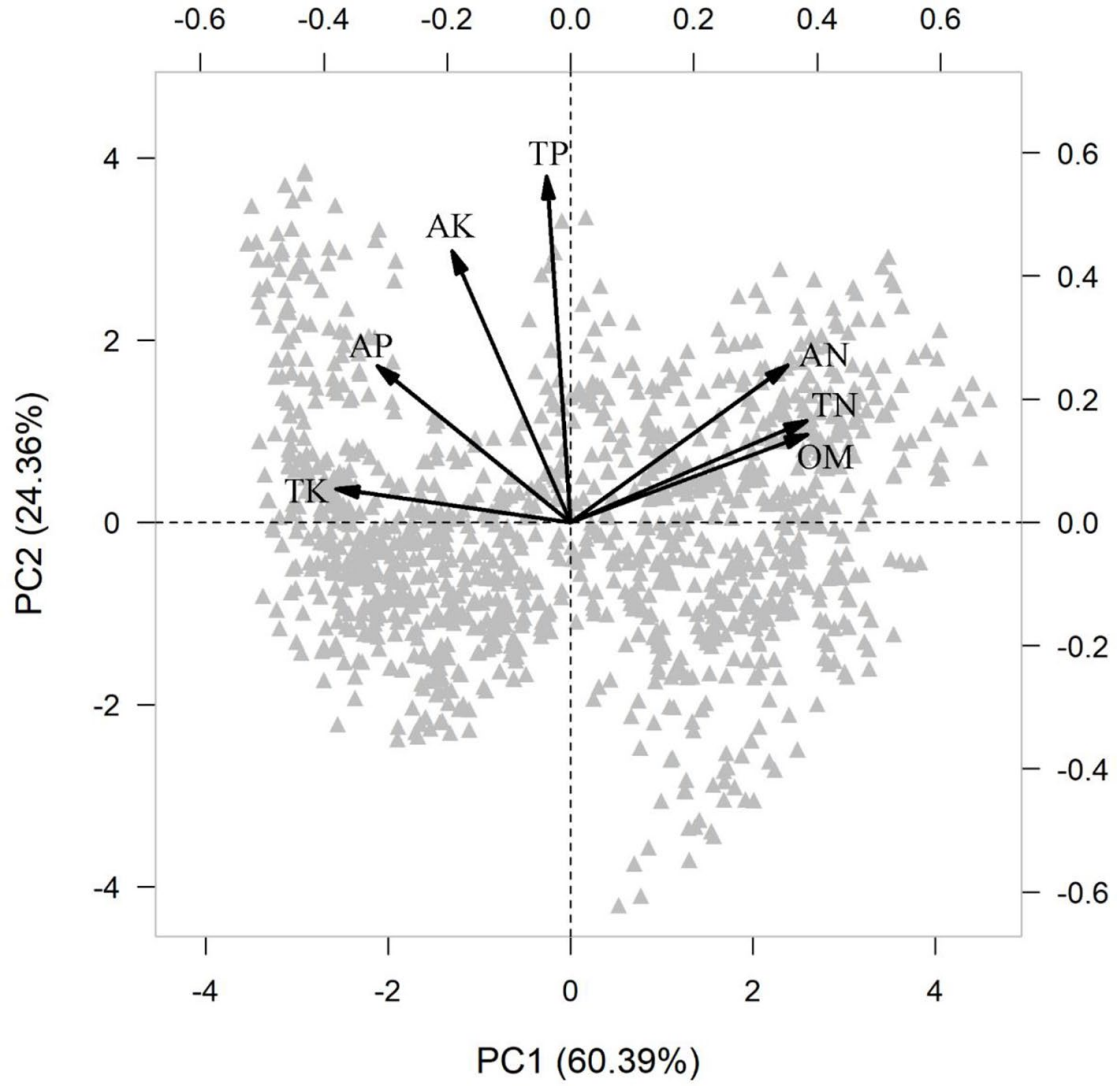
Principal component analysis (PCA) of measures of soil fertility across 1158 seedling plots. Soil variable included organic matter (OM, %), available nitrogen (AN, mg g^-1^), total nitrogen (TN, mg g^-1^), available phosphorus (AP, mg g^-1^), total phosphorus (TP, mg g^-1^), available potassium (AK, mg g^-1^) and total potassium (TK, mg g^-1^).

In the PCA of the various measures of soil fertility, the first principal component (PC1) captured 60% of the variation (**Table S4** and **Figure 2**), representing the soil fertility gradient from high organic matter (OM) and total and available nitrogen (TN, AN) together with low available phosphorus (AP) and total potassium (TK), to the other extreme with low OM, TN, and AN together with high AP and TK.

Species that invest in cheap tissue were expected to be associated with fast resource-acquisition strategies and resource-rich environments, whereas investment in expensive tissue were expected to be associated with resource conservation strategies and resource-poor environments (**Figure 1**), so that species with a fast resource acquisition strategy should have relative higher LA, LAR, LN, LP, SLA, SSL, RBI, RN, RP, SRA, SRL and lower LC, LDMC, T, DIAM, RTD, and be found more often in habitats with high soil fertility and light.

**Figure 3:**
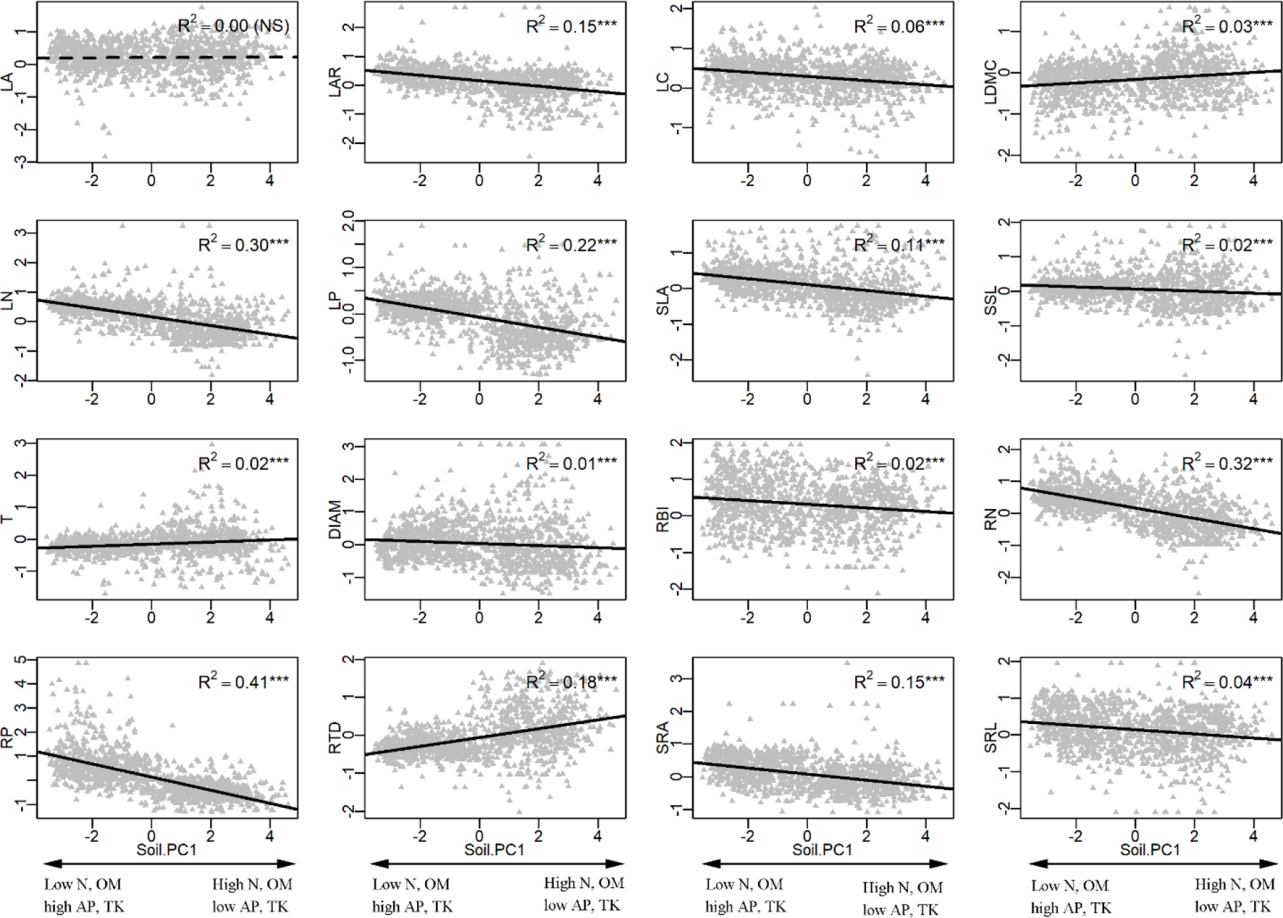
Univariate linear regression analyses between soil fertility (soil PC1) and the community-weighted mean (CWM) of plant traits. ***P < 0.001. See **Table 1** and **Figure 1** for trait and soil fertility abbreviations, respectively.

**Figure 4:**
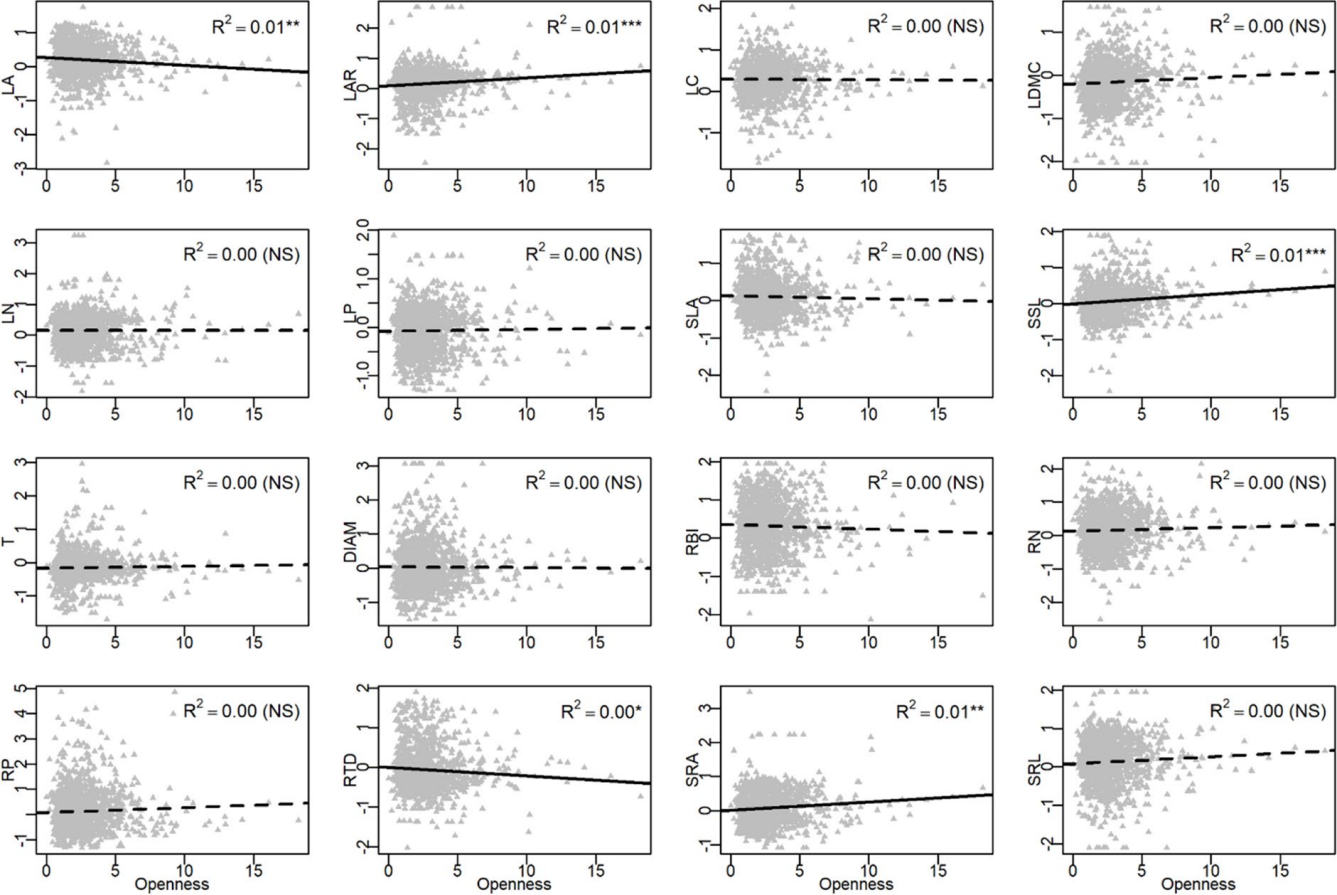
Univariate linear regression analyses between canopy openness and the community-weighted mean (CWM) of plant traits. See **Table 1** for trait abbreviations. *P < 0.05, **P < 0.01, ***P < 0.001.

In the univariate linear regression model, the community weighted mean (CWM) trait was highly correlated with soil fertility (**Figure 3**) and all traits from different organs, excepted for LA, were significantly correlated with soil PC1. Nitrogen and phosphorus content of leaves and roots were best predicted by soil fertility, with 22% to 41% of variation explained by soil PC1. The relationships between traits and soil fertility were consistent with expectation (**Figure 1**), except for LC-soil PC1 and DIAM-soil PC1. For example, traits that are positively associated with a resource-acquisition strategy (e.g., SLA, LN, RN, and SRL) were all negatively correlated with soil PC1; and traits that are positively associated with resource conservation, such as LDMC, T and RTD, were all positively associated with soil PC1. Soil PC2 represents a gradient of total phosphorus and available potassium (**Figure 2**), of which high levels were also related to species with a fast resource-acquisition strategy (**Figure S4**). Plant traits were less often correlated with canopy openness (**Figure 4**). However, several PES traits showed significant correlation with canopy openness; LAR, SSL, and SRA was positively connected to openness, and LA and RTD were negatively correlated.

**Figure 5:**
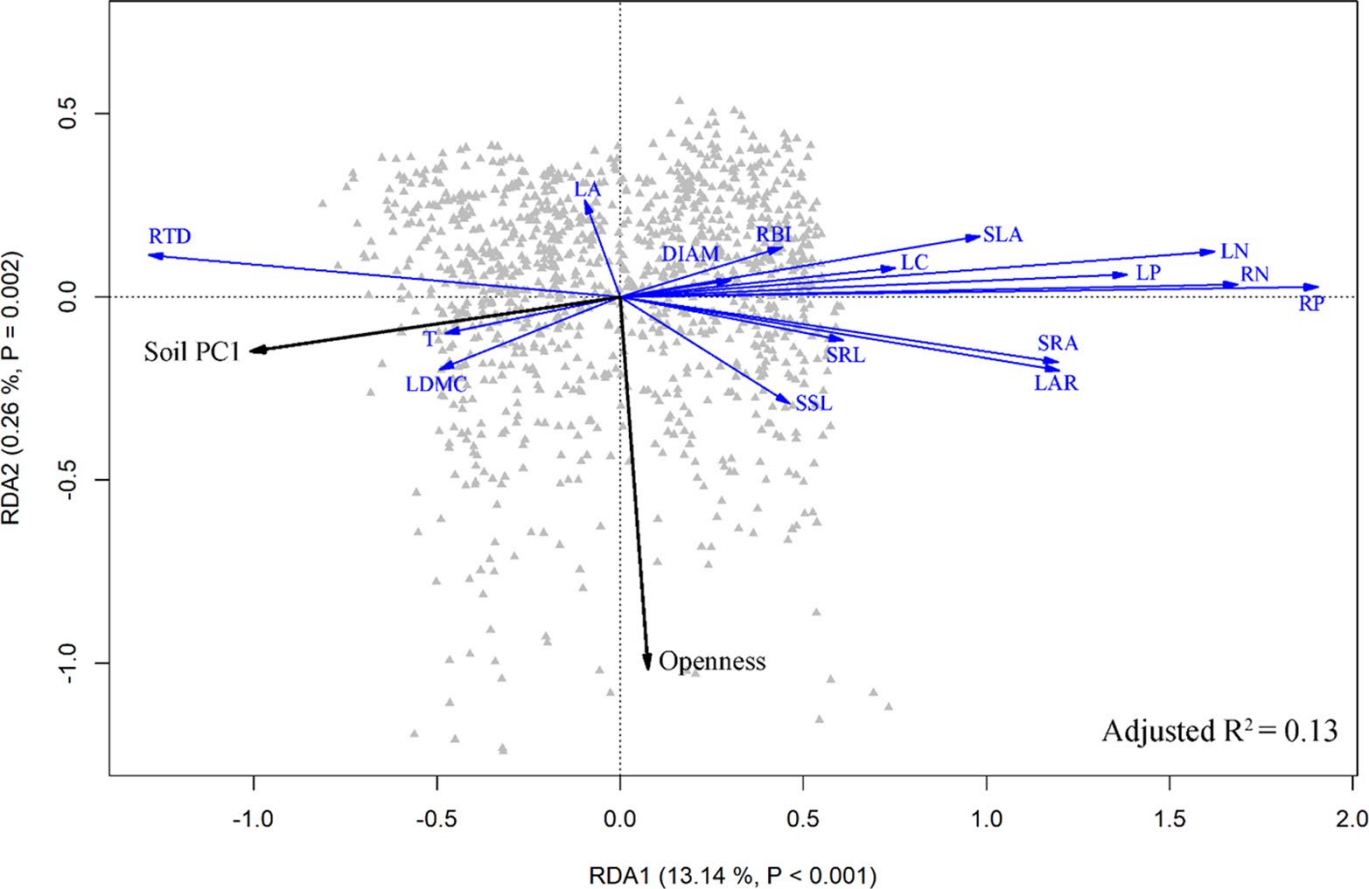
Ordination triplot of redundancy analysis (RDA) of the community-weighted mean (CWM) of plant traits. Black arrows indicate environmental factors, and blue arrows were traits. See **Table 1** for trait abbreviations.

The result of the RDA summarized the inter-correlation among traits, and trait-environment relationships (**Figure 5**). Most traits from aboveground organs and roots reflected variation along the whole-plant economics spectrum, in which species on the high end of the first RDA axis (right side) had relative higher LAR, LN, LP, SLA, SSL, RBI, RN, RP, SRA, SRL and low LDMC, T, RTD, representing the fast resources acquisition strategy of species. Species at the left side tended to be resource conservation strategists. Soil PC1 was loaded on the left side of the first RDA axis (**Figure 5**), indicating that traits were strongly correlated with soil fertility, and species with resource-conservation strategies were associated with high organic matter and nitrogen, and with low phosphorus and potassium (**Figure 2**). Canopy openness tended to be loaded on the second RDA axis, indicating that openness was weakly related to soil fertility and traits.

## Discussion

This study demonstrated that aboveground and belowground traits of plants tended to be coordinated, aboveground traits were good predictors of root traits, and traits from different organs showed corresponding patterns of adaptation along the gradients of soil fertility and light. Species that presented resource-acquisition traits were associated with environments with high soil P, soil K, and canopy openness plus low N and organic matter; plant species with traits adapted for resource conservation were instead associated with environments with the inverses of those environmental characteristics (**Figure 5**). Light environment and soil fertility were the focus of our study because they are considered the most limiting resources in tropical and subtropical forests (Uriarte et al., 2005; Wright et al., 2011; Pasquini and Santiago, 2012; Santiago et al., 2012; Record et al., 2016; Han et al., 2017). However, other environmental conditions also have the potential to drive functional responses of plants.

### Aboveground Traits Are Good Predictors of Root Traits

One of the main predictions from the plant economics spectrum is that traits from different organs should be coordinated (Reich, 2014); this suggests that root traits should be predictable from known aboveground traits. We found, for 57 species of subtropical tree seedlings, that aboveground and root traits were highly correlated (**Table 2**), and that the signs of the correlations were congruent across organs (see expected relationships in **Figure 1**), highlighting the existence of a plant economics spectrum for this subtropical seedling community. Previous studies have found similar patterns in other systems: N, P, and dry matter content of roots, leaves, and stems were positively correlated (Freschet et al., 2010; Geng et al., 2014), plants with low leaf or root N content tended to also have high leaf and root tissue density (Craine et al., 2005; Perez-Ramos et al., 2012), SRL was positively correlated with SLA (Freschet et al., 2015), and RTD and LDMC were positively correlated (Bergmann et al., 2017). However, traits of root diameter (DIAM) and branching intensity (RBI) seem to be independent of the whole-plant economics spectrum, highlighting the multidimensionality of root traits (Kramer-Walter et al., 2016; Weemstra et al., 2016). This points to the need to incorporate the complexity of the soil environment, root form and function, and multiple belowground resource uptake strategies (e.g., mycorrhizal pathways) in future studies (Weemstra et al., 2016).

We identified several aboveground traits that are most useful as predictors of corresponding root traits and evaluated the strength of the associations (**Table 3**). Predictive power reached up to 22–40% for PES root traits (excluding DIAM and RBI) only using 1–3 aboveground traits, confirming a moderately useful predictive potential of aboveground traits when direct measurement of root traits is unavailable. Overall, leaf area ratio (LAR) and leaf nitrogen (LN) were the best predictors of PES root traits in this subtropical forest.

However, the relationships among traits in different organs may vary across environmental gradients. Craine et al. (2005) found that relationships between leaf and root traits were stronger at regional than global scales, and that environmental factors such as soil properties might affect their relationships (Craine et al., 2005). Others noted that the SLA-SRL relationship could shift from negative to positive with increasing temperature (Geng et al., 2014); LN_area_/RN_length_ increased and SLA/SRL and LN_mass_/RN_mass_ decreased from semi-arid to arid environments (Liu et al., 2010). These results demonstrate that uncovering the trait relationships among different plant organs should be linked to environmental gradients, especially for large-scale studies.

### Limitation by Soil Fertility Is the Main Factor Driving the Functional Response

Another fundamental prediction of plant economics spectrum theory is that resource-associated traits from different organs should show adaptive congruence along resource gradients (Reich, 2014). Species with fast resource acquisitive strategy should be more abundant in fertile soil, especially for the key soil nutrients (e.g., N, P, and K concentration) (Lu et al., 2010; Wright et al., 2011; Pasquini and Santiago, 2012; Santiago et al., 2012; Record et al., 2016). In our study, we used a 10-year census of 1158 seedling plots to examine this hypothesis. Traits tended to vary predictably along the soil PC1 axis, indicating that limitation by soil fertility was the main ecological factor driving the functional response (Perez-Ramos et al., 2012). Our results showed that nutrient-acquisitive species were more abundant at the low end of the soil PC1 axis (**Figures 3** and **5**), where soils had high phosphorus and potassium but low nitrogen and organic matter (**Figure 2**). This contrasts with results from previous studies, where many traits did not show adaptive patterns along resource gradients. For example, fast-growing, nutrient-acquisitive species were unexpectedly more abundant in soils with high N or P (Freschet et al., 2010; Orwin et al., 2010); low plant tissue density (e.g., leaf dry matter content or root tissue density) corresponded to soils with high N, but SRA increased with increasing soil C:N ratio (Perez-Ramos et al., 2012). In addition, soil nutrient (N, P, K) availability increased LAR, SLA, but decreased SRL (Freschet et al., 2015); increasing soil P and decreasing C:N ratio were associated with decreasing RTD, RBI and SRL but increasing RN and DIAM (Kramer-Walter et al., 2016). We suggest that multidimensional traits may lead to these mixed results, because some traits fall outside of the plant economics spectrum, implying that these traits are constrained by environmental drivers that are not necessarily related to resource uptake (Weemstra et al., 2016), resulting in weak or inconsistent relationships with environments.

However, although plant traits show clear patterns of adaptive matching along the resource gradient represented by the soil PC1 axis, our results do not fully support the expectations of the plant economics spectrum. We note signs of resource conservation in resource-poor environments and resource acquisition in resource-rich environments (**Figure 1**), a clear trade-off between high nitrogen and organic matter on one end and phosphorus and potassium at the other (**Figure 2**), and resource-acquisitive species are more abundant in the soil high in P and K but low in N and OM (**Figures 3** and **S4**). Because limiting resources should be the main ecological factors that drive the functional response of plants (Perez-Ramos et al., 2012), our results suggest that P and K may be the most limiting soil nutrients in this subtropical forest, so that species adapted for resource acquisition were more successful in the environments where those nutrients were abundant. Previous studies came to similar conclusions that P was the most limited element in plant growth in old, weathered soils of tropical forests (Walker and Syers, 1976; Condit et al., 2013; Liu et al., 2018) and that K addition increased photosynthesis rates and height growth of tropical seedlings (Pasquini and Santiago, 2012; Santiago et al., 2012).

### Functional Traits Response to Canopy Openness

Besides soil nutrients, seedling response to light could be another important axis that differentiates niches; previous results showed that seedlings are limited by light in the shaded understory of tropical and subtropical forests (Uriarte et al., 2005; Han et al., 2017) and variation in ecophysiological traits of trees are expected to help capture the variation in light (Reich et al., 1998; Poorter and Rose, 2005; Sanchez-Gomez et al., 2006; Coble et al., 2017). In our study, however, functional traits showed much weaker response to canopy openness than to soil fertility (**Figure 5**). Only 5 out of 16 traits showed a significant relationship with canopy openness (**Figure 4**). These traits did show patterns of adaptation along a light gradient, where species with traits adapted for fast resource acquisition (e.g., high LAR, SSL, SRA, and low RTD) were more abundant in the seedling plots with greater canopy openness, consistent with the PES expectation (**Figure 1**). There are at least two factors that could lead to the relatively weak response of traits to canopy openness. First, light in most of the seedling plots was consistently very low (**Table S1**), with >90% of seedling plots <5% canopy openness. There were very few canopy gaps associated with plots in this study, resulting in low spatial heterogeneity of light availability and corresponding difficult to measure trait-based responses among plants (Record et al., 2016). Second, we did not measure temporal heterogeneity of light, since we only measured canopy openness in 2017; unidentified temporal variation in canopy openness could also contribute to the weak relationship between plant traits and the light environment.

### Biomass Allocation Plays an Important Role in Responding to Environments

Both leaf area ratio (LAR) and specific stem length (SSL) are traits involve in biomass allocation, determining how much leaf area or stem length is produced per unit of plant mass (Poorter et al., 2012). Both these traits were associated with gradients of soil fertility and canopy openness (**Figures 3** and **4**), indicating that plants adapt to different environments not only through organ morphology but through variation in biomass allocation (Freschet et al., 2015). For example, shifts in biomass allocation were important in plant responses to soil nutrients (Freschet et al., 2015).

However, some relationships between biomass allocation, traits, and environments in our study were not consistent with previous results and expectations (**Figure 1**). For example, Freschet et al. (2015) found that increased soil nutrient supply drives an increase in SLA, leaf mass fraction (LMF, leaf dry mass/total plant dry mass), and LAR, but a decrease in SRL, root mass fraction (RMF, root dry mass/total plant dry mass), and root length ratio (RLR, root length/total plant dry mass) (Freschet et al., 2015). While increased light supply drives a decrease in SLA, LMF, and LAR (Reich et al., 1998), it increases RMF. In our study, SLA, SRL, LAR, and SSL all increased with increasing soil P and K and with decreasing N and OM; increasing light availability also increased LAR and SSL. We suggest that these relationships may derive from the specific environments of tropical and subtropical seedlings; where light and soil nutrients were extremely limited in the understory in these regions (Uriarte et al., 2005; Lu et al., 2010; Wright et al., 2011; Pasquini and Santiago, 2012; Santiago et al., 2012; Record et al., 2016; Han et al., 2017), all variation in organ morphology and biomass allocation are coordinated to best use the limited resources. For example, in response to increased availability of P, K, and light, seedling species with a fast resource acquisition strategy not only increased SLA and SRL to enhance the resource acquisition but also increased biomass allocation to leaf area (LAR) and height (SSL) to achieve a relative high resource use efficiency and growth rate.

## Conclusion

The plant economics spectrum helps explain ecological strategies, community assembly, and ecosystem functioning (Reich, 2014), but empirical evidence is still limited to evaluate how broadly applicable these patterns are across diverse systems. Our study provides evidence for a plant economics spectrum in a subtropical seedling community, presents the potential to extrapolate from readily measured aboveground traits to more inaccessible belowground plant functions, and suggests that limitation by soil fertility is the main factor driving the functional response in this forest. We found that both variation in organ morphology and biomass allocation play important roles in responding to local environments. These results provide insights into how plant ecological strategies shape community assembly and responses of plant communities to environmental change. The understanding of plant strategies will continue to improve by combining large scale studies with more species and measured traits (e.g. tree height and production) in the future.

## Data Availability Statement

The datasets generated for this study are available on request to the corresponding author.

## Author Contributions

SY and YS conceived the project. YS, WL, MF, and HL collected the data. YS, GG, and WL analyzed the data. SY, YS, and GG wrote the first draft of the manuscript. All authors contributed to manuscript revision.

## Funding

The study was funded by the National Natural Science Foundation of China [31830010 and 31500338], the Natural Science Foundation of Guangdong Province [2017A030313199], the National Key Research and Development Program of China [Project No. 2017YFA0605100].

## Conflict of Interest

The authors declare that the research was conducted in the absence of any commercial or financial relationships that could be construed as a potential conflict of interest.
