# Supplementary material for: Linking Aboveground Traits to Root Traits and Local Environment: Implications of the Plant Economics Spectrum
Source: Front Plant Sci. 2019 Oct 30;10:1412. doi: 10.3389/fpls.2019.01412 (PMC6831723; doi:10.3389/fpls.2019.01412)
Supplement: Supplementary file 1 [file DataSheet_1.docx]

**Supporting Information**

**Linking aboveground traits to root traits and local environment: implications of the plant economics spectrum**

Yong Shen^1^, Gregory S. Gilbert^2^, Wenbin Li^1^, Miao Fang^1^, Huanping Lu^3^ and Shixiao Yu^1*^

^1^Department of Ecology, School of Life Sciences/State Key Laboratory of Biocontrol, Sun Yat-sen University, Guangzhou 510275, PR China

^2^Department of Environmental Studies, University of California, Santa Cruz, CA 95064, U.S.A.

^3^Guangdong Ecological Meteorology Center, Guangzhou 510080, PR China

Author for correspondence:

Shixiao Yu

Tel: +86 20 39332980

Email: lssysx@mail.sysu.edu.cn

**Table S1.** Summary of the environmental factors for 1158 seedling plots included in this study. Measurement methods and references are showed in the table

| Environmental factor of seedling plot | Unit | Mean | Median | Minimum | Maximum | Measurement method | Reference |
| --- | --- | --- | --- | --- | --- | --- | --- |
| Organic matter | mg g^-1^ | 60.51 | 59.19 | 27.34 | 143.89 | Potassium dichromate oxidation method | ([Sun et al., 2009](#_ENREF_3)) |
| Total nitrogen | mg g^-1^ | 2.01 | 1.99 | 1.07 | 3.62 | Kjeldah method | ([de Castilho et al., 2006](#_ENREF_1)) |
| Available nitrogen | mg g^-1^ | 0.18 | 0.18 | 0.10 | 0.29 | Alkaline hydrolysis pervasion method | ([Yu et al., 2014](#_ENREF_5)) |
| Total phosphorus | mg g^-1^ | 0.12 | 0.12 | 0.08 | 0.17 | Molybdenum-antimony colorimetry | ([Wang et al., 2014](#_ENREF_4)) |
| Available phosphorus | mg g^-1^ | 0.0036 | 0.0032 | 0.0008 | 0.0115 | Molybdenum-antimony colorimetry | ([Wang et al., 2014](#_ENREF_4)) |
| Total potassium | mg g^-1^ | 24.29 | 26.48 | 2.15 | 48.86 | Flame atomic absorption spectrophotometry | ([de Castilho et al., 2006](#_ENREF_1)) |
| Available potassium | mg g^-1^ | 0.09 | 0.09 | 0.05 | 0.18 | Flame atomic absorption spectrophotometry | ([de Castilho et al., 2006](#_ENREF_1)) |
| Canopy openness | % | 2.83 | 2.49 | 0.02 | 18.23 | Digital camera, WinSCANOPY | ([Record et al., 2016](#_ENREF_2)) |

**Table S2.** Summary of the 16 functional traits for 57 species included in this study

| Trait | Mean | Median | Minimum | Maximum | Coefficient of  variation (%) | Unit |
| --- | --- | --- | --- | --- | --- | --- |
| LA | 19.25 | 16.73 | 1.63 | 60.46 | 0.61 | cm^2^ |
| LAR | 69.43 | 68.25 | 24.57 | 181.79 | 0.41 | cm^2^ g^-1^ |
| LC | 40.64 | 40.85 | 33.42 | 47.05 | 0.07 | % |
| LDMC | 0.37 | 0.37 | 0.14 | 0.51 | 0.22 | g g^-1^ |
| LN | 18.05 | 16.96 | 10.72 | 40.14 | 0.29 | mg g^-1^ |
| LP | 0.55 | 0.50 | 0.29 | 1.05 | 0.32 | mg g^-1^ |
| SLA | 203.04 | 193.04 | 78.13 | 427.28 | 0.38 | cm^2^ g^-1^ |
| SSL | 34.86 | 33.48 | 9.56 | 75.71 | 0.45 | cm g^-1^ |
| T | 0.17 | 0.15 | 0.07 | 0.35 | 0.34 | cm |
| DIAM | 0.54 | 0.52 | 0.33 | 0.99 | 0.25 | mm |
| RBI | 1.62 | 1.63 | 0.75 | 2.58 | 0.27 | tips cm^-1^ |
| RN | 12.95 | 11.96 | 2.27 | 30.24 | 0.42 | mg g^-1^ |
| RP | 0.76 | 0.61 | 0.24 | 4.39 | 0.77 | mg g^-1^ |
| RTD | 0.40 | 0.38 | 0.16 | 0.67 | 0.32 | g cm^-3^ |
| SRA | 233.95 | 212.74 | 116.50 | 637.76 | 0.37 | cm^2^ g^-1^ |
| SRL | 1569.07 | 1487.55 | 540.82 | 3835.86 | 0.45 | cm g^-1^ |

Traits include leaf area (LA), leaf area ratio (LAR), leaf carbon content (LC), leaf dry matter content (LDMC), leaf nitrogen content (LN), leaf phosphorus content (LP), specific leaf area (SLA), stem specific length (SSL), leaf thickness (T), fine root average diameter (DIAM), root branching intensity (RBI), root nitrogen content (RN), root phosphorus content (RP), root tissue density (RTD), specific root area (SRA) and specific root length (SRL)

**Table S3.** Test of phylogenetic signal (Blomberg’s K) of 16 functional traits across 57 plant species, calculated using the raw trait scores, standardized (subtracting the mean and dividing by the standard deviation), or log-transformed

|  | Trait-raw | | Trait-standardized | | Trait-logged | |
| --- | --- | --- | --- | --- | --- | --- |
|  | *K* | P value | *K* | P value | *K* | P value |
| LA | 0.32 | 0.07 | 0.32 | 0.08 | 0.18 | 0.86 |
| LAR | 0.29 | 0.14 | 0.29 | 0.15 | 0.24 | 0.31 |
| LC | ***0.44*** | ***0.001*** | ***0.44*** | ***0.003*** | ***0.46*** | ***0.001*** |
| LDMC | ***0.45*** | ***0.001*** | ***0.45*** | ***0.002*** | ***0.38*** | ***0.01*** |
| LN | ***0.39*** | ***0.02*** | ***0.39*** | ***0.02*** | ***0.47*** | ***0.001*** |
| LP | ***0.40*** | ***0.01*** | ***0.40*** | ***0.003*** | ***0.35*** | ***0.01*** |
| SLA | ***0.40*** | ***0.002*** | ***0.40*** | ***0.008*** | ***0.40*** | ***0.001*** |
| SSL | 0.24 | 0.39 | 0.24 | 0.38 | 0.20 | 0.74 |
| T | 0.23 | 0.49 | 0.23 | 0.49 | 0.22 | 0.50 |
| DIAM | ***0.70*** | ***0.001*** | ***0.70*** | ***0.001*** | ***0.72*** | ***0.001*** |
| RBI | ***0.57*** | ***0.001*** | ***0.57*** | ***0.001*** | ***0.57*** | ***0.001*** |
| RN | ***0.36*** | ***0.01*** | ***0.36*** | ***0.01*** | ***0.38*** | ***0.01*** |
| RP | 0.43 | 0.10 | 0.43 | 0.11 | 0.29 | 0.12 |
| RTD | ***0.40*** | ***0.001*** | ***0.40*** | ***0.004*** | ***0.36*** | ***0.01*** |
| SRA | 0.24 | 0.44 | 0.24 | 0.45 | 0.22 | 0.53 |
| SRL | ***0.35*** | ***0.01*** | ***0.35*** | ***0.03*** | 0.29 | 0.11 |

Significant phylogenetic signal was highlighted in bold and italic. See Table S2 for trait abbreviations

**Table S4.** Factor loadings of the first two components of principal component analysis (PCA) on soil fertilities, explaining 84.75% variation in soil fertilities

| Soil fertility | PC1 (60.39%) | PC2 (24.36%) |
| --- | --- | --- |
| Organic matter (OM, %) | 0.46 | 0.17 |
| Available nitrogen (AN, mg g^-1^) | 0.42 | 0.31 |
| Total nitrogen (TN, mg g^-1^) | 0.46 | 0.20 |
| Available phosphorus (AP, mg g^-1^) | -0.37 | 0.30 |
| Total phosphorus (TP, mg g^-1^) | -0.05 | 0.68 |
| Available potassium (AK, mg g^-1^) | -0.23 | 0.53 |
| Total potassium (TK, mg g^-1^) | -0.45 | 0.07 |


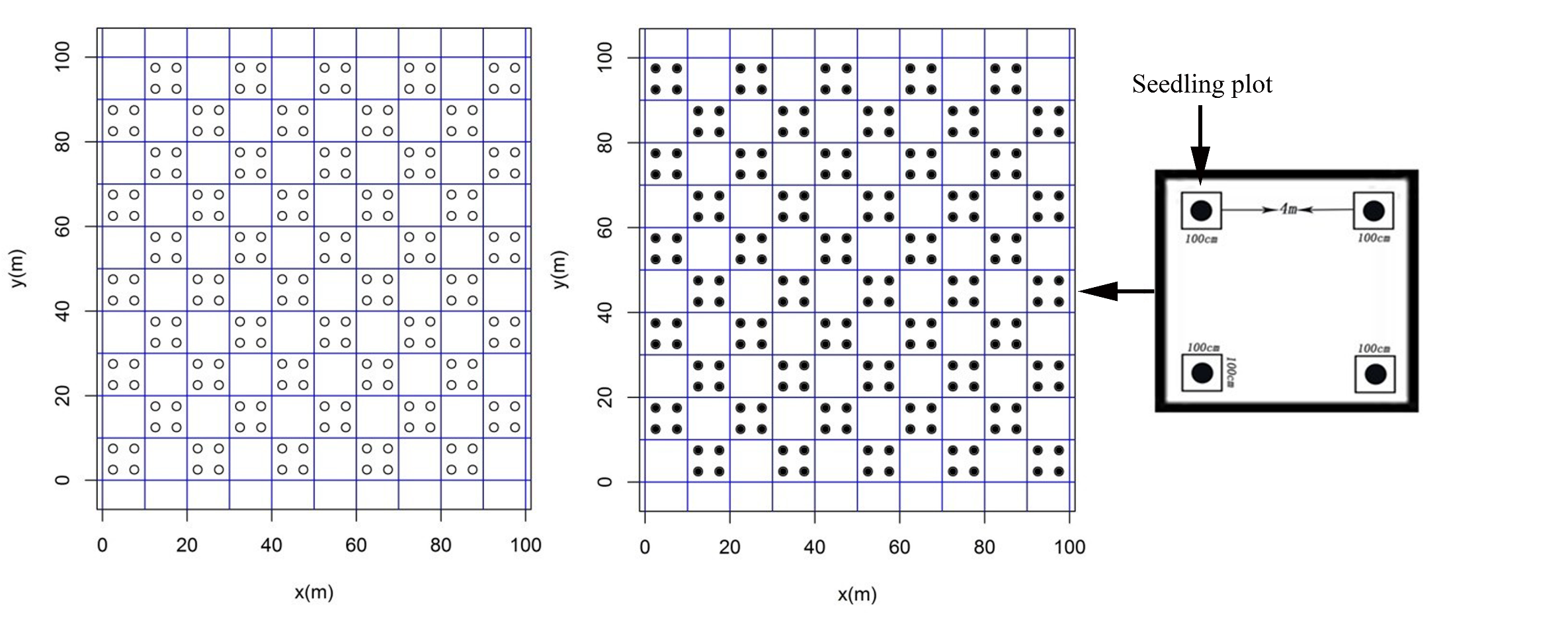


**Figure S1.** The distribution of seedling plots in the 1-ha permanent plot in Heishiding Nature Reserve. Two types of regular distribution were showed. Each of the six 1-ha plots was randomly assigned one of these two checkerboard patterns


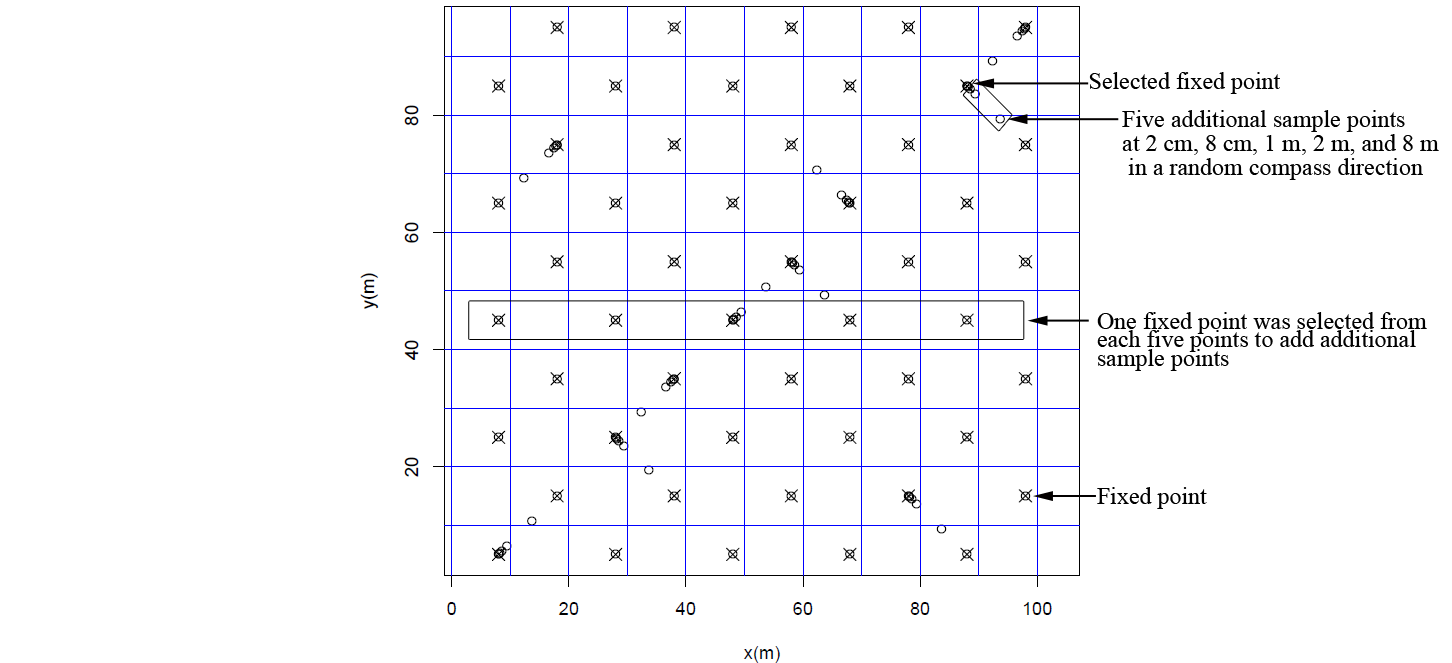


**Figure S2.** Soil samples were collected at 50 fixed points in a grid across the 1-ha permanent plot in Heishiding Nature Reserve. In addition, one fixed point was selected from each row of the grid for additional sampling; at each of the 10 selected fixed points a random compass direction (N, E, S, W, NE, NW, SE or SW), was selected, and then additional soil samples collected at 2 cm, 8 cm, 1 m, 2 m, and 8 m from the fixed point (50 additional samples for a total of 100 soil samples across the 1-ha plot). This was replicated across each of the six ha for a total of 600 soil samples


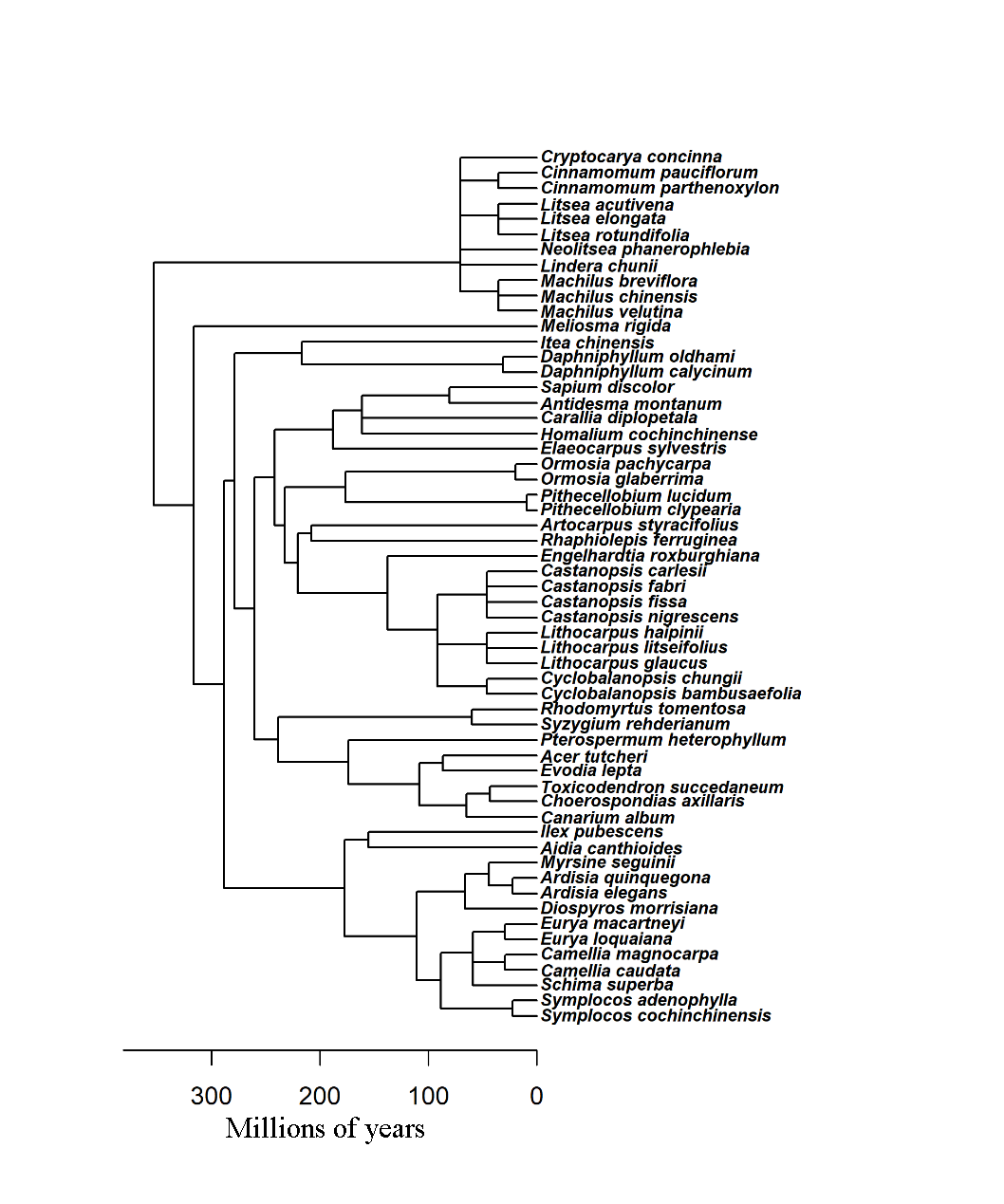


**Figure S3.** Phylogenetic tree of the 57 seedling species included in the study.


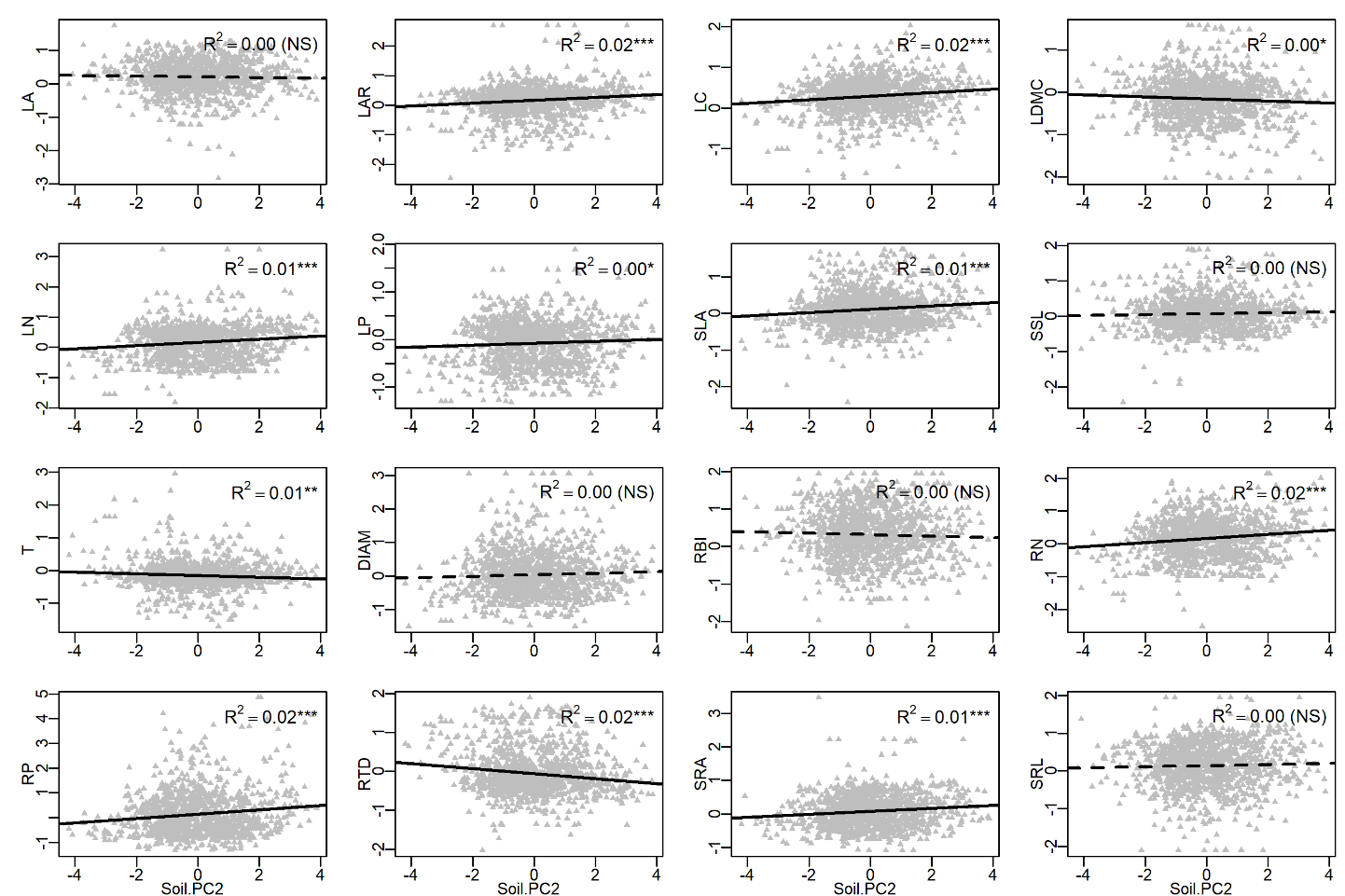


**Figure S4.** Univariate linear regression analyses between soil fertilities (soil PC2, represented high total phosphorus and available potassium) and the community-weighted mean (CWM) of plant traits. *** P < 0.001. See Figure 2 for principal component analysis (PCA) and Table S2 for trait abbreviations.

**References**

de Castilho, C.V., Magnusson, W.E., de Araujo, R.N.O., Luizao, R.C.C., Lima, A.P., and Higuchi, N. (2006). Variation in aboveground tree live biomass in a central Amazonian Forest: Effects of soil and topography. *Forest Ecology and Management* 234(1-3)**,** 85-96. doi: 10.1016/j.foreco.2006.06.024.

Record, S., Kobe, R.K., Vriesendorp, C.F., and Finley, A.O. (2016). Seedling survival responses to conspecific density, soil nutrients, and irradiance vary with age in a tropical forest. *Ecology* 97(9)**,** 2406-2415. doi: 10.1002/ecy.1458.

Sun, G., Luo, P., Wu, N., Qiu, P.F., Gao, Y.H., Chen, H., et al. (2009). Stellera chamaejasme L. increases soil N availability, turnover rates and microbial biomass in an alpine meadow ecosystem on the eastern Tibetan Plateau of China. *Soil Biology & Biochemistry* 41(1)**,** 86-91. doi: 10.1016/j.soilbio.2008.09.022.

Wang, G.Y., Zhang, S.R., Xu, X.X., Li, T., Li, Y., Deng, O.P., et al. (2014). Efficiency of nanoscale zero-valent iron on the enhanced low molecular weight organic acid removal Pb from contaminated soil. *Chemosphere* 117**,** 617-624. doi: 10.1016/j.chemosphere.2014.09.081.

Yu, F.K., Huang, X.H., Duan, C.Q., He, S.Z., Zhang, G.S., Liu, C.E., et al. (2014). Impacts of Ageratina adenophora invasion on soil physical-chemical properties of Eucalyptus plantation and implications for constructing agro-forest ecosystem. *Ecological Engineering* 64**,** 130-135. doi: 10.1016/j.ecoleng.2013.12.050.
